# Similar cognitive deficits in mice and humans in the chronic phase post-stroke identified using the touchscreen-based paired-associate learning task

**DOI:** 10.1038/s41598-020-76560-x

**Published:** 2020-11-11

**Authors:** Wei Zhen Chow, Lin Kooi Ong, Murielle G. Kluge, Prajwal Gyawali, Frederick R. Walker, Michael Nilsson

**Affiliations:** 1 School of Biomedical Sciences and Pharmacy and Priority Research Centre for Stroke and Brain Injury, University of Newcastle, University Drive, Callaghan, NSW 2308 Australia; 2 Hunter Medical Research Institute, New Lambton, Heights, NSW Australia; 3 NHMRC Centre of Research Excellence Stroke Rehabilitation and Brain Recovery, Heidelberg, VIC Australia; 4 Centre for Rehab Innovations, University of Newcastle, Callaghan, NSW Australia; 5 LKC School of Medicine, Nanyang Technological University, Novena Campus, Singapore; 6 School of Pharmacy, Monash University Malaysia, Bandar Sunway, Selangor Malaysia; 7 School of Health and Wellbeing, Faculty of Health, Engineering and Sciences, University of Southern Queensland, Queensland, Australia

**Keywords:** Spatial memory, Stroke

## Abstract

For many chronic stroke survivors, persisting cognitive dysfunction leads to significantly reduced quality of life. Translation of promising therapeutic strategies aimed at improving cognitive function is hampered by existing, disparate cognitive assessments in animals and humans. In this study, we assessed post-stroke cognitive function using a comparable touchscreen-based paired-associate learning task in a cross-sectional population of chronic stroke survivors (≥ 5 months post-stroke, n = 70), age-matched controls (n = 70), and in mice generated from a C57BL/6 mouse photothrombotic stroke model (at six months post-stroke). Cognitive performance of stroke survivors was analysed using linear regression adjusting for age, gender, diabetes, systolic blood pressure and waist circumference. Stroke survivors made significantly fewer correct choices across all tasks compared with controls. Similar cognitive impairment was observed in the mice post-stroke with fewer correct choices compared to shams. These results highlight the feasibility and potential value of analogous modelling of clinically meaningful cognitive impairments in chronic stroke survivors and in mice in chronic phase after stroke. Implementation of validated, parallel cross-species test platforms for cognitive assessment offer the potential of delivering a more useful framework for evaluating therapies aimed at improving long-term cognitive function post-stroke.

## Introduction

Chronic stroke survivors often experience persistent memory and learning impairment in multiple cognitive domains^1–4^. One of the challenges in advancing research on cognitive impairment post-stroke is to establish and validate test platforms that allow for parallel cognitive assessments in humans and animals in experimental models of stroke^5^. This was recently reiterated in the core recommendations from the recent second Stroke Recovery and Rehabilitation Roundtable^6^.

The Cambridge Neuropsychological Test Automated Battery (CANTAB) is a frequently used, well-validated touchscreen-based tool utilised in assessment of multi-domain cognitive function in neurodegenerative disorders such as Alzheimer’s disease^7^. It has, for example, enabled early detection of mild cognitive impairment with high sensitivity and specificity, and accurately predicted progression to Alzheimer’s disease in a prospective cohort^8^. In stroke, CANTAB has, so far, only been used to assess the visual and spatial working memory in sub-acute patients at two weeks post-stroke^9–11^.

In rodent models of stroke, spatial memory and learning are most frequently assessed using animal behavioural tasks, for example the Morris water maze and novel object recognition^12,13^. These tasks share only limited similarities with clinical assessments of cognitive function in stroke survivors. Further, results derived from these tasks are often quite variable, which could be due to aversive nature of the tasks and experimenter interference/interpretation^14,15^. Application of rodent touchscreen operant platforms allows an adapted form of CANTAB paired-associate learning (PAL) assessment to be conducted in mouse models of stroke with comparable assessment of cognitive processes and mode of delivery (via an automated touchscreen platform) to that which can be used to assess cognition in stroke survivors. Furthermore, two prior studies demonstrated the feasibility of aligning touchscreen-based cognitive tests in mice and humans carrying mutations in the postsynaptic Discs large homolog (*Dlg*) gene family, using comparable tasks, specifically the CANTAB PAL in humans and rodent PAL task in mice^16^, and an identical visuospatial PAL task between species^17^. Both studies identified clinically relevant cognitive impairment in Schizophrenia patients and mice using parallel touchscreen-based assessments^16,17^. Despite strong incentives, a similar cross-species comparison is still to be made between rodents and humans in chronic phase after stroke.

Our group has previously reported on the use of the rodent touchscreen platform to assess cognitive decline in mice after photothrombotic stroke^18–20^. We demonstrated that the rodent touchscreen platform constitutes a pertinent assessment tool which is sensitive to the effects of pharmacological/therapeutic interventions to improve cognitive function after stroke^19–21^. The major question that we wanted to address was “do mice and humans in the chronic phase post-stroke respond similarly when evaluated using touchscreen delivered PAL task?” The aim of the present study was to apply and in parallel evaluate the touchscreen-based visuospatial object-location PAL task in chronic stroke survivors and in mice in chronic phase after experimental stroke.

## Materials and methods

### Study population

A total of 140 participants were recruited between November 2017 and February 2019 in NSW, Australia. Stroke survivors in the chronic phase of stroke recovery (≥ 5 months post-stroke) were recruited via the Hunter Stroke Research Volunteer Register (HSRVR) based at the Hunter Medical Research Institute (HMRI). Control participants without a history of stroke were recruited from both HMRI control registry and via social media advertisements. Exclusion criteria included a history of pituitary and adrenal gland diseases. The participants were part of a cross-sectional study that was designed to examine the association of stress and resiliency with functional outcome among stroke survivors^22,23^.

### Assessment of visuospatial PAL function in humans

All participants completed a CANTAB Motor Screening task at the beginning of the cognitive assessment to determine whether sensorimotor deficits or lack of comprehension will limit the collection of valid data from the participant. Cognitive performance was assessed in all participants using the CANTAB visuospatial PAL task^7^. There were four stages (consisting of 2, 4, 6 and 8 patterns) to be completed. Boxes were displayed on the screen and opened randomly to reveal a unique pattern in one or more boxes. The patterns were then displayed in the middle of the screen, one at a time, and the participant must touch the box where the pattern was originally located. If the participant makes an error, the patterns are re-presented to remind the participant of their locations. PAL performance was assessed based on the overall correct choices made at the first attempt of recalling the correct patterns and their locations (PALFAMS), overall total errors made (adjusted), total number of attempts made and total number of patterns reached at the last stage of the task (Table 2). We obtained an effect size, d of 0.55 using retrospective power calculation of PALFAMS.

### Animal experimental design

#### Sample size estimation

Sample size was estimated using the formula^24^ to compare the mean values of cognitive performance in PAL between sham and stroke groups:

$${\text{Sample size}} = \frac{{2{\text{SD}}^{2} { }\left[ {\left( {z_{1 - \alpha /2} } \right) + \left( {z_{1 - \beta } } \right)} \right]^{2} }}{{d^{2} }}$$

Using preliminary data of the percentage of correct rate (primary outcome) achieved by sham mice in PAL, we obtained a standard deviation, SD of 6.146 and an effect size, d of 0.25. Allowing a type 1 error of 5%, α = 0.05 with the power of 90%, β = 0.1 we calculated a sample size of seven animals per group. A sample size of more than seven animals per group will ensure that the effect of stroke on the measured outcome can be measured with a greater than 90% chance. A total of 45 C57BL/6 male mice aged 7–8 weeks old were obtained from the Animal Services Unit at the University of Newcastle. All mice were housed in standard cages provided with food and water ad libitum in a temperature (21 °C ± 1) and humidity-regulated environment under a 12:12 h reverse light–dark cycle (lights on at 19:00). Mice were acclimatised to the experimenter and housing during the first week before they were randomly subjected to sham or stroke surgery. At 20 weeks post-stroke, cognitive function was assessed in 24 mice (sham, n = 12 and stroke, n = 12) using PAL task which involved daily assessment for an hour/session, five days a week for a total of 35 sessions. For histological analysis, mice (sham, n = 8 and stroke, n = 12) without behavioural testing was euthanized at 27 weeks post-stroke.

### Photothrombotic occlusion

Photothrombotic occlusion was performed as described previously with minor modifications^19,25^. Briefly, mice were anaesthetized by 2% isoflurane during the surgical procedure on a temperature-regulated (37 °C ± 1) stereotaxic frame. A total of 200 µl of Rose Bengal (Sigma-Aldrich, USA), a photosensitive dye, was injected intraperitoneally at 10 mg/ml in sterile saline in mice subjected to stroke. Saline was injected intraperitoneally in sham. The skull was exposed by incision of the skin along the midline of the scalp. After eight minutes, the exposed skull was illuminated for 15 min by a 4.5 mm diameter cold light source, a well-established procedure in our laboratory, which previously have been shown to induce reproducible deficits in paired-associative learning in the mice^19^.

### Assessment of visuospatial PAL performance in mice

At 19 weeks post-stroke, 24 mice (sham, n = 12 and stroke, n = 12) were introduced to a series of habituation and training tasks performed in the Bussey-Saksida Mouse Touch Screen Chamber (Campden Instruments Ltd, UK) for five days as described previously^16,17,19,20,26^. Mice were randomly assigned to behavioural testing by a researcher blinded to the animal’s treatment group. Four mice (3 sham and 1 stroke mice) failed to achieve the criteria during the training period and were excluded from the PAL assessment. An hour of PAL training session comprised of maximum 36 tasks, in which the session is completed when either the time limit or completion of all tasks was reached. All mice performed PAL sessions during five consecutive days for seven weeks in total. PAL performance was assessed based on the correct rate, number of correction trials, time taken to complete a session, number of tasks completed per session, correct or incorrect touch latency and reward collection latency.

### Statistical analyses

In the human study, non-parametric tests were used to analyse non-normal continuous outcomes data. Linear regression was performed to analyse the effect of stroke on cognitive performance, with adjustment for known confounders including age, gender, diabetes, systolic blood pressure and waist circumference^27^. A generalised linear regression model with robust standard errors was used to analyse the association in non-normally distributed residuals. In mice, PAL data was analysed in mean values of five sessions for a total of seven blocks using two-way ANOVA and Sidak multiple comparisons. Brain tissue volume data was analysed using two-sided t-test. *p* < 0.05 values were considered statistically significant. All analyses were conducted in SPSS v21 (IBM Corp, USA) and Prism for Windows v7.02 (GraphPad Software, USA).

### Ethical approval

The clinical study was approved by the Hunter New England Human Research Ethics Committee of HNE Local Health District (reference 17/06/21/4.02). This study was registered with the Australian New Zealand Clinical Trials Registry (ACTRN12617000736347) and can be found at (https://www.anzctr.org.au/Trial/Registration/TrialReview.aspx?id=372896). All experiments were performed in accordance with relevant guidelines and regulations for studies involving humans according to the Declaration of Helsinki. All participants provided written informed consent to participate in the study. Each study participant also provided written consent regarding utilisation of information and images related to the study for publication purpose, if required. Animal experiments were approved by the University of Newcastle Animal Care and Ethics Committee (A-2013-338), and performed in accordance with the ARRIVE guidelines^28^. All animal experiments were also conducted in accordance with the New South Wales Animals Research Act (1985) and the Australian Code of Practice for the use of animals for scientific purposes.

## Results

In the current cohort, 58.6% (n = 41) of stroke survivors had ischemic stroke and 37.1% (n = 26) had haemorrhagic stroke. The remaining 4.3% (n = 3) was of unknown origin (Table 1). The time post-stroke [median (IQR)] in the study population was 38.5 (13.8 to 117.5) months. The mean age was not significantly different between the two groups (stroke, 61.9 (13.8) years vs control, 64.6 (10.0) years, *p* = 0.192). The gender distribution was significantly different between stroke and non-stroke control cohorts (*p* = 0.027). In the control cohort, 65.7% (n = 46) were females compared to 45.7% (n = 32) in the stroke cohort. Dyslipidaemia was reported in 54.3% (n = 38) of the stroke cohort compared to 22.9% (n = 16) in the control cohort (*p* < 0.001). It should be noted that the details of stroke were self-reported by the study participants, and the primary measure for this cohort was to investigate the relationships between stress and resilience with functional outcomes in long-term survivors of stroke, as reported in our recent publication^22,23^.

**Table 1:** Baseline characteristics of the study population.

Characteristics	Stroke, n = 70	Non-stroke control, n = 70	*P* value
Age (years), mean (SD)	61.9 (13.8)	64.6 (10.0)	0.192
Gender, n (%)			
Male	38 (54.3)	24 (34.3)	**0.027**
Female	32 (45.7)	46 (65.7)	
Types of stroke, n (%)			
Ischemic	41 (58.6)	N/A	
Haemorrhagic	26 (37.1)	N/A	
Unknown	3 (4.3)	N/A	
Systolic blood pressure (mmHg), mean (SD)	131 (17)	131 (18)	0.985
Diastolic blood pressure (mmHg), mean (SD)	78 (12)	79 (6)	0.724
BMI, mean (SD)	29.01 (6.3)	28.0 (5.7)	0.332
Waist circumference (cm), mean (SD)	98.7 (21.5)	95.4 (15.5)	0.301
Diabetes, n (%)	10 (14.3)	6 (8.6)	0.234
Hypertension, n (%)	28 (40.0)	21 (30.0)	0.131
Dyslipidaemia, n (%)	38 (54.3)	16 (22.9)	**< 0.001**

Statistically significant (*p* < 0.05) results are bolded.

The human participants were assessed using the CANTAB visuospatial PAL task (Table 2). CANTAB PAL data in humans (all analysed parameters) was not normally distributed (*p* < 0.05 in Shapiro–Wilk test). Hence, the non-parametric Mann–Whitney U test was used to compare ranks between stroke survivors and controls. The PAL task for human consisted of four levels of difficulty. Difficulty levels one, two, three and four respectively consisted of the two, four, six and eight object patterns. PALFAMS assessed the overall correct choices made by the participants at the first attempt of recalling the correct patterns and their locations. Stroke survivors made significantly lower total number of correct choices at their first attempt of recalling the correct patterns compared to the control participants (PALFAMS: stroke 9.0 (6.0, 11.0) Vs Control 11.0 (8.0, 14.0); *p* = 0.002). Unadjusted linear regression showed a significant inverse association between stroke and the first attempt memory scores (crude β (95% CI), − 2.26 (− 3.62 to − 0.899), *p* = 0.001). The association remained significant after adjusting for potential confounders (adj β =  −2.47 (− 3.85 to − 1.09), *p* = 0.001).

**Table 2:** Comparison of CANTAB PAL performance in stroke survivors and controls.

Measure name	Description	Stroke, n = 70	Non-stroke control, n = 70	P-value
PALFAMS	PAL First Attempt Memory Score. The number of times a participant chose the correct box on their first attempt when recalling the pattern locations. Calculated across all assessed trials	9.0 (6.0, 11.0)	11.0 (8.0, 14.0)	**0.002**
PALTA2	PAL Total Attempts 2 Patterns. The total number of attempts made (but not necessarily completed) by the participant during assessment problems containing a total of 2 shapes to recall	1.0 (1.0, 1.0)	1.0 (1.0, 1.0)	**< 0.001**
PALTA4	PAL Total Attempts 4 Patterns	2 (1.0, 3.0)	1.5 (1.0, 3.0)	**0.016**
PALTA6	PAL Total Attempts 6 Patterns	3 (2.0, 4.0)	3 (2.0, 4.0)	0.472
PALTA8 -	PAL Total Attempts 8 Patterns	4 (3.0, 4.0)	4 (2.0, 4.0)	0.143
PALTA	PAL Total Attempts Overall (cumulative count across all assessed trials)	10.0 (8.0, 12.0)	9 (7.7, 11.0)	**0.015**
PALTEA2	PAL Total Errors 2 Shapes. The number of times the participant chose the incorrect box for a stimulus on assessment problems, where the number of shapes required to remember was equal to 2, plus an adjustment for the estimated number of errors they would have made on any other 2 pattern problems, attempts and recalls they did not reach	0.0 (0.0, 0.0)	0.0 (0.0, 0.0)	**< 0.001**
PALTEA4	PAL Total Errors 4 Shapes	3.0 (0.0, 5.0)	0.5 (0.0, 3.0)	**0.008**
PALTEA6	PAL Total Errors 6 Shapes	4.5 (3.0, 12.0)	2.0 (4.5, 10.0)	0.302
PALTEA8	PAL Total Errors 8 Shapes	16.5 (8.0, 28.0)	12.5 (5.75, 28.0)	0.86
PALTEA	PAL Total Errors Overall (cumulative count across all assessed trials)	24.0 (14.0, 43.75)	21.0 (8.0, 38.25)	**0.044**

Data are presented in median (inter-quartile range) for numerical variables. All *p* values < 0.05 are considered statistically significant (bolded).

PALTA assessed the total number of attempts made by participants overall as well as at each difficulty level. A maximum of four attempts was allowed at each level and if the participants did not make a correct choice within the given four attempts, they were not prompted to higher difficulty level and the task was terminated. The rate of dropout due to unsuccessful fourth attempt was higher among stroke survivors compared to controls after each level (0, 3, 7 and 24 vs. 0, 0, 5 and 18). However, the difference in the rate of dropout did not reach statistical significance at any level. The highest number of possible attempts (i.e. four) was recorded for both stroke survivors and controls for the difficulty level they did not reach. This adjustment provided an opportunity to compare the number of attempts stroke survivors and controls made in order to pass the specific difficulty level. Stroke survivors required a significantly greater number of attempts to pass difficulty level one (two patterns, *p* < 0.001) and two (four patterns, *p* = 0.016) when compared to controls. The cumulative count of attempts across all assessed trials were significantly higher among stroke survivors compared to controls (*p* = 0.015).

The data for total errors made by participants at each difficulty level and overall was automatically adjusted (PALTEA) by CANTAB by providing a value for the probable number of errors participants would made at the unattempted level. Stroke survivors made higher number of errors overall (*p* = 0.044), and at difficulty level one (two patterns, *p* < 0.001) and two (four patterns, *p* = 0.008) compared to controls.

**Figure 1:**
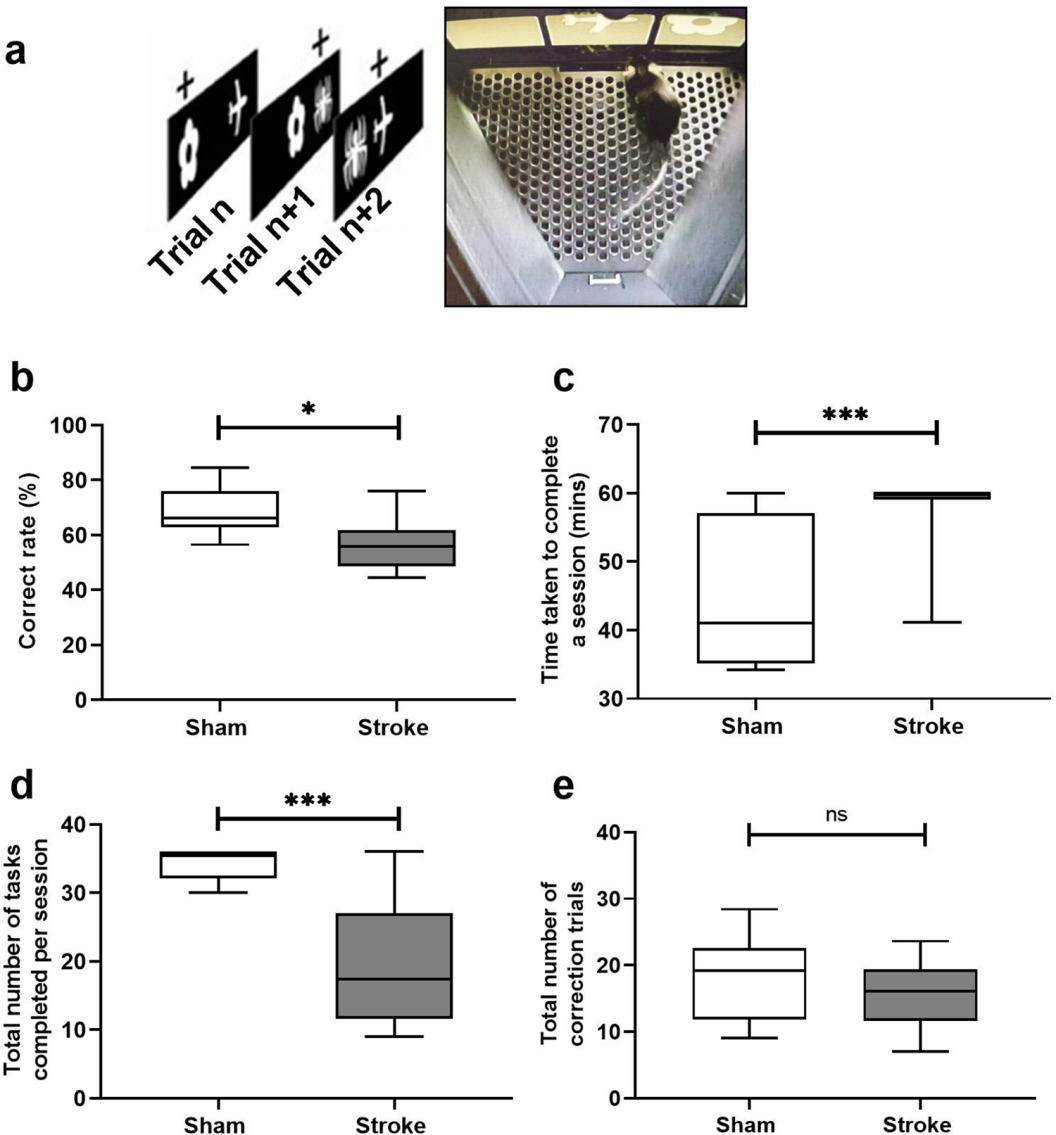
Mouse model of stroke tested on a rodent touchscreen-based object-location paired-associates learning (PAL) task. (**a**) Representative image of a mouse performing a comparable PAL task on a rodent touchscreen operant platform at 6 months post-stroke (sham = 9, stroke = 11)^35^. (**b**) Correct rate (%). (**c**) Time taken to complete a session (mins). (**d**) Total number of tasks completed per session. (**e**) Total number of correction trials. Data in mean ± SD shown for PAL performance at 6 months post-stroke (block 7) and represents an average of five consecutive sessions per block. *p* < 0.05 (*), and *p* < 0.001 (***) values were indicated where applicable.

At six months post-stroke in mice, confirmation of stroke was determined by the presence of significant tissue loss in the ipsilesional (IL) hemisphere and corpus callosum using histological analyses (Supplementary Fig. S1). We also confirmed that there was a significant sustained neuronal loss and persistent reactive astrogliosis in the peri-infarct (Supplementary Fig. S2). The mice were assessed using the rodent touchscreen visuospatial PAL task (Fig. 1a). Similarly, mice subjected to stroke performed worse in object-location PAL with a significantly lower correct rate compared to sham (stroke, 57.1 ± 9.0% vs sham, 69.1 ± 8.7%, *p* = 0.032) (Fig. 1b). There was a significant effect of stroke (F(1,18) = 5.65; *p* = 0.029) and time (F(6,108) = 15.1; *p* < 0.0001) on the mean correct rate. Sham mice demonstrated a progressive acquisition of PAL during the assessment while mice post-stroke had consistently performed at 50% (chance level) (Supplementary Fig. S3a). Sham mice required less time to complete 36 tasks by the last block of sessions (week 7) and were able to complete more tasks (Fig. 1c,d). Mice subjected to stroke required significantly longer time to complete a session (stroke, 57.7 min ± 5.67 vs sham, 46.1 min ± 10.9, *p* < 0.0001), and performed reduced number of tasks (stroke, 19.3 tasks ± 8.82 vs. sham, 34.1 tasks ± 2.32, *p* < 0.0001). Together, these findings indicate impaired visuospatial PAL in mice subjected to stroke. However, we did not observe significant changes in the total number of correction trials between stroke and sham mice (stroke, 15.5 trials ± 4.66 vs. sham, 19.5 trials ± 5.80, *p* = 0.702) (Fig. 1e). The mean latency time taken to make a correct touch, incorrect touch or reward collection did not differ significantly between stroke and sham mice (Supplementary Fig. S3e–g).

## Discussion

In the current study we evaluated the performance of both mice and humans on the comparable PAL task in chronic phase post-stroke. Using a highly analogous assessment platform we observed that it was possible to detect and compare PAL deficits post-stroke in *both* humans and mice. To the best of our knowledge, this is the first time this cross-species comparison has been made in chronic phase after stroke.

More than 56% of stroke survivors report cognitive dysfunction at six months post-stroke^1^. Cognitive impairment post-stroke has been identified in multiple cognitive domains, including executive functions, language, verbal and visual PAL and motor skills^2,3,29^. Furthermore, stroke-induced cognitive dysfunction will continue to evolve in a large proportion of the stroke survivors over subsequent years post-stroke^2^. Consistent with these reports, we observed that stroke survivors suffered from persistent cognitive impairment of the visuospatial PAL many years after stroke. Previously, attention and visuospatial functions at three months after stroke have been shown to be independently associated with reduced quality of life at one year post-stroke^30^. Current understanding of the mechanisms underpinning long-term cognitive recovery post-stroke are limited. Despite accumulating data from animal studies, clinical translation of novel therapies to improve cognitive function in stroke survivors in the chronic phase has been hampered, at least partly, due to disparities of the methods for cognitive assessment in mice and humans^5^. Cognitive assessment using CANTAB and other similar platforms have been extensively used to consider changes in cognition post-stroke in human^9–11^. However, no studies to date have considered whether similar deficits using similar assessment methodologies would be observed in rodents post-stroke.

Characterising a comparable cognitive assessment method in humans and mice constitutes a preliminary step towards narrowing the translational gap to improve our understanding of cognitive impairment post-stroke. To date, three studies had targeted cognitive dysfunction in other pathological conditions, using parallel touchscreen-based approach^16,17,31^. In two studies, the authors aimed to understand cognitive decline linked to mutations in the *Dlg* gene family in humans and mice, demonstrating successful parallel assessment of PAL using the touchscreen platforms^16,17^. Furthermore, parallel touchscreen-based assessments have recently been applied to characterise clinically relevant motivational deficits in Huntington’s disease patients and mice^31^. In the present study, we assessed the visuospatial PAL in both chronic stroke survivors and mice in chronic phase post-stroke using CANTAB and rodent touchscreen platforms, respectively. CANTAB PAL task assesses a person’s visuospatial episodic memory and is very sensitive to hippocampal function^7^. We demonstrated that the participants (mixed stroke subtype background) had impaired visuospatial PAL characterised by an overall reduced number of correct choices across all tasks and increased overall errors made compared to controls.

In rodents, comparable visuospatial PAL assessments adapted on the touchscreen operant platform have been well-validated in animal models of disease such as Alzheimer’s disease^26,32–35^, neuropsychiatric disorders^16,17^ and, more recently by our group, in stroke^19,20^. The rodent findings had shown that PAL performance was sensitive to hippocampal lesions, and manipulations of the cholinergic and glutamatergic systems^26,33^. We previously demonstrated the utility of rodent PAL task in the evaluation of pharmacological/therapeutic interventions aimed at improving cognitive function in mice after photothrombotic stroke^19,20^. The rodent PAL task shares at least two similarities with the CANTAB PAL assessment in humans; both tasks measure similar forms of cognitive process (visuospatial PAL and is sensitive to hippocampal function) and can be administered via a similar mode (touchscreen platform). These advantageous characteristics enable an identical approach to visuospatial PAL assessment in humans and mice. Some differences exist between the two versions of PAL tasks (summarised in Table 3). However, these differences have not impeded a parallel assessment of similar cognitive process in both humans and mice. Several patterns can be presented in the human CANTAB PAL assessment (with a maximum of eight object patterns). We found that the two object patterns PAL assessment used in humans was most comparable with the rodent PAL task since both versions required encoding and retrieval processes of two novel stimuli during the test and effectively detected cognitive impairment in humans and mice post-stroke. In principle, an extensive habituation/training period was a required integral part of the touchscreen-based cognitive assessment in rodents compared with humans. The characteristics and location of the stimuli were constant throughout rodent PAL assessment with a constant level of task complexity, which could also have induced practice effects in the animals. On the other hand, CANTAB PAL comprised of at least four different stages of PAL with increasing task complexity and randomisation of the number, location and characteristics of stimuli which may have reduced potential practice effects in humans.

**Table 3:** Comparability between the touchscreen-based human CANTAB paired-associates learning (PAL) and rodent version of PAL task.

Characteristics	CANTAB PAL	Rodent PAL
Type of cognitive process assessed	Visuospatial episodic memory and is sensitive to hippocampal function	
Mode of delivery	Touchscreen	
Overall measure of correct choices	First attempt memory score	Rate of correct choices (%)
Overall measure of errors	Mean errors to success and total errors (adjusted)	Number of correction trials performed
Duration of assessment	Cross-sectional	Longitudinal
Pre-training/habituation prior to assessment	No pre-training is required	A series of habituation tasks is required
Training length (mins)	Eight	60
Number of tasks per session	Four tasks, completion of each task activates the following task	36
Number of objects (visual stimuli) presented at one time	Two, four, six or eight patterns	Two patterns
Criteria for completion of task	Either the time limit is reached or the maximum number of patterns had been correctly selected	Either the time limit is reached or the maximum number of tasks had been completed (including both correct and incorrect choices)
Reward for correct choices	None	Strawberry-flavoured milkshake
Task complexity	Increasing task complexity, but modifiable	Constant task complexity, but modifiable
Practice effects	Randomisation of number, location and characteristics (colours and shapes) of stimuli reduces possible practice effects	Standardised presentation of three different stimuli (flower, plane and spider), each with a specific location across all tasks may cause possible practice effects

Since this was a cross-sectional study, we did not assess pre-stroke cognition which is one of the known confounders of cognitive function post-stroke. Furthermore, the sample population of stroke survivors represented a wide range of stroke subtypes and time post-stroke which, most likely, would have led to different inter-individual cognitive performance. For this cohort, we neither had access to the study participants’ clinical demographics describing the infarct characteristics (such as the location and number of infarcts) nor the educational background, which are known determinants of cognitive function post-stroke^10^. In the animal experiments, motor function was not assessed post-stroke. However, upon presentation of the patterns on the touchscreen, the duration to make a choice required by both stroke and sham groups (correct/incorrect) or collect the reward from dispenser (for correct choice) were not significantly different across all sessions. The potential influence of post-stroke mouse motor function on the PAL test should be assessed in future studies.

In conclusion, our findings bring new insights to the evaluation process of cognitive decline in chronic stroke survivors using identical cross-species test platforms for analogous assessments in humans and animals. Through this comparative approach, we should be able to expand our understanding of post-stroke cognitive decline leading to development of novel therapies aimed at improving post-stroke cognitive impairment.

## Supplementary information

Supplementary information.
