# Supplementary material for: Similar cognitive deficits in mice and humans in the chronic phase post-stroke identified using the touchscreen-based paired-associate learning task
Source: Sci Rep. 2020 Nov 11;10:19545. doi: 10.1038/s41598-020-76560-x (PMC7658221; doi:10.1038/s41598-020-76560-x)
Supplement: Supplementary file 1 — Supplementary information. [file 41598_2020_76560_MOESM1_ESM.docx]

**Title page**

**Title:** Similar cognitive deficits in mice and humans in the chronic phase post-stroke identified using the touchscreen-based paired-associate learning task

**Authors and affiliations:** Wei Zhen Chow^1,2,3^, Lin Kooi Ong^1,2,3,4,6^, Murielle Kluge^1,2^_,_ Prajwal Gyawali^1,2^, Frederick R. Walker^1,2,4*^, Michael Nilsson^1,2,3,4,5*^.

*Contributed equally to senior authorship

1 School of Biomedical Sciences and Pharmacy and Priority Research Centre for Stroke and Brain Injury, University of Newcastle, Callaghan, NSW, Australia.

2 Hunter Medical Research Institute, New Lambton, Heights, NSW, Australia.

3 NHMRC Centre of Research Excellence Stroke Rehabilitation and Brain Recovery, Heidelberg, VIC, Australia.

4 Centre for Rehab Innovations, University of Newcastle, Callaghan, NSW, Australia.

5 LKC School of Medicine, Nanyang Technological University, Singapore.

6 School of Pharmacy, Monash University Malaysia, Bandar Sunway, Selangor, Malaysia.

**Address for correspondence:**

Michael Nilsson, University of Newcastle, University Drive, Callaghan, NSW 2308, Australia.

Phone: +61 2 4042 0570 Email: Michael.Nilsson@newcastle.edu.au

Frederick Rohan Walker, University of Newcastle, University Drive, Callaghan, NSW 2308, Australia.

Phone: +61 4 1611 0434 Email: Rohan.Walker@newcastle.edu.au

**Supplementary Methods**

**Brain tissue extraction and processing**

All mice were euthanized at 27 weeks post-stroke with 200 µl sodium pentabarbitol and the brains were extracted. Briefly, for histology analysis, the mice were transcardially-perfused with cold sterile saline followed by cold 4 % paraformaldehyde (PFA) at pH 7.4 for 12 minutes^1^. PFA-fixed brains were post-fixed in 4 % PFA with 12.5 % sucrose solution followed by storage in 0.1M phosphate buffer solution (PBS) with 12.5 % sucrose at 4 °C. Fixed brains were sectioned at 30 µm on a freezing microtome (LEICA SM2010R, Leica Biosystems) and stored in antifreeze solution in a 24-well plate at 4 °C until further analysis. For protein analysis, the mice were transcardially-perfused with cold sterile saline. Brain were dissected and rapidly frozen. Fresh frozen brains were sectioned at 200 µm on a cryostat, and peri-infarct territories were obtained for protein extraction and western blotting.

**Nissl body staining for tissue volume analysis**

For the analysis of IL and contralesional (CL) hemispheric tissue volume, a total of seven rostral-caudal free-floating 30 µm sections (bregma +1.5 to -2.5) was selected for each mouse brain (sham, n=8 and stroke, n=12), and stained using 0.1 % cresyl violet solution (#C5042, Sigma, USA) as described previously^1^. For the analysis of specific regional tissue volume, three brain sections per region representing the forceps minor, corpus callosum and hippocampus were selected at a distance of 0.18 mm apart in each brain (sham, n=9 and stroke, n=12). Tissue volume in both IL and CL regions were measured using the formula: area of the region (mm^2^) x thickness of the interval between the sections (0.18 mm). The ratio of IL:CL volume was computed for each region using the formula: tissue volume in the IL ÷ tissue volume in the CL region. Data was analysed using the ImageJ software 1.50 a (NIH, USA).

**Protein extraction and western blotting**

Protein extraction and western blotting of the peri-infarct region were performed as described^2^. Primary antibodies used include mouse anti-Aldh1L1 (1:2000, #MABN495, Millipore, USA), mouse anti-GFAP (1:5000, #12943, CST, USA), and rabbit anti-NeuN (1:2000, #3670, CST). Secondary antibodies used include anti-mouse-HRP antibody (1:10000, Biorad, USA), and rabbit IgG (1:7500, Biorad). Anti-β-actin-HRP (1:50000, #A3854, Sigma-Aldrich, USA) was used as a loading control. Membranes were visualized on an Amersham Imager 600 (GE Healthcare, USA) using Luminata Classico Western HRP substrate (Millipore, USA), and the band densities were measured using the Amersham Imager 600 analysis software.

**Supplementary Figures**


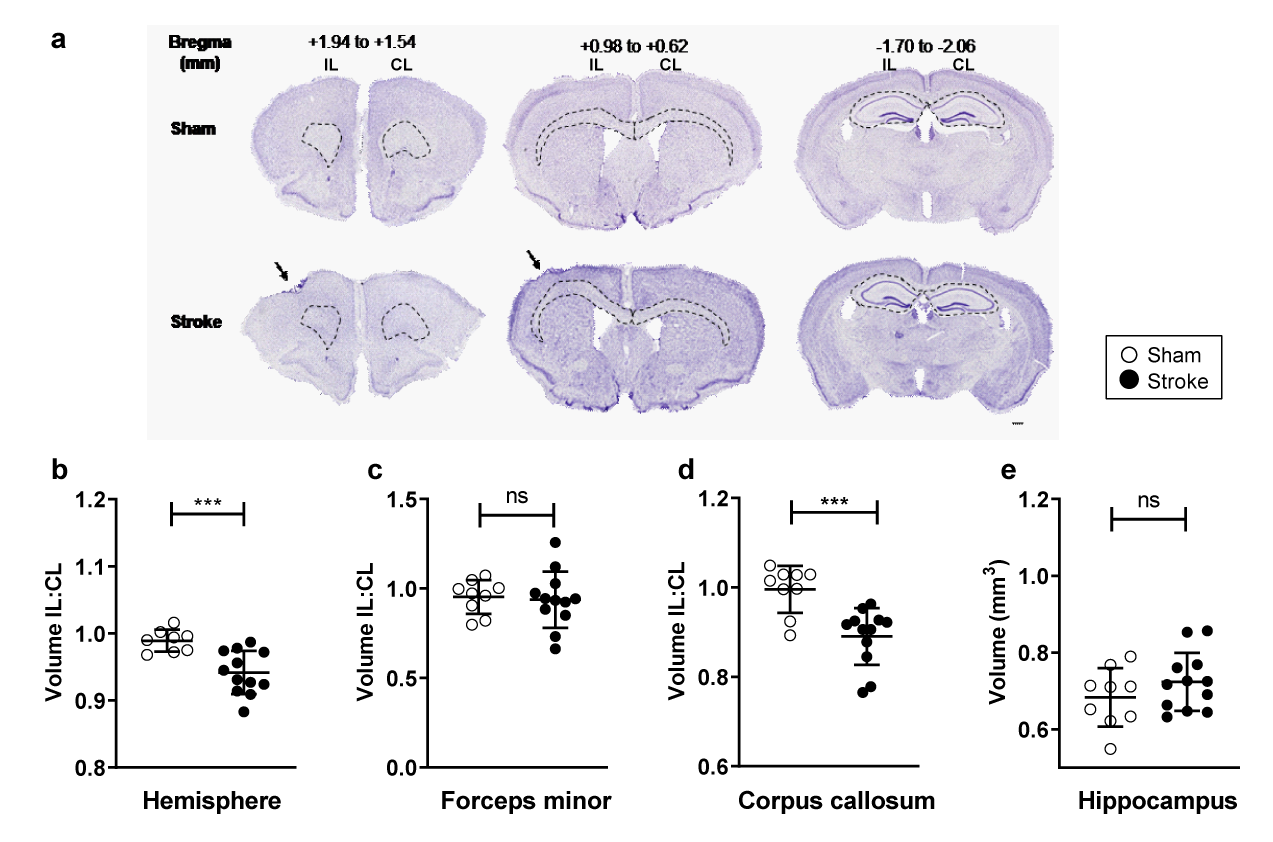


**Supplementary Fig. S1.** **Histological assessment of mouse brain tissue volume at six months post-stroke.** **a**. Representative images of selected brain sections stained with Nissl stain. Arrows highlight the location of infarct. The dotted lines indicate the tissue area measured in the forceps minor, corpus callosum and hippocampus. **b**. IL:CL brain hemispheric volume in stroke mice compared to sham. Images were shown at 20x magnification. Scale bar, 300 µm. **c,** Volume IL:CL of forceps minor. **d.** Volume IL:CL of corpus callosum. **e.** Volume of hippocampus. IL, ipsilesional; CL, contralesional. Data were presented in mean ± SD (sham=8, stroke=12). p<0.001 (***)


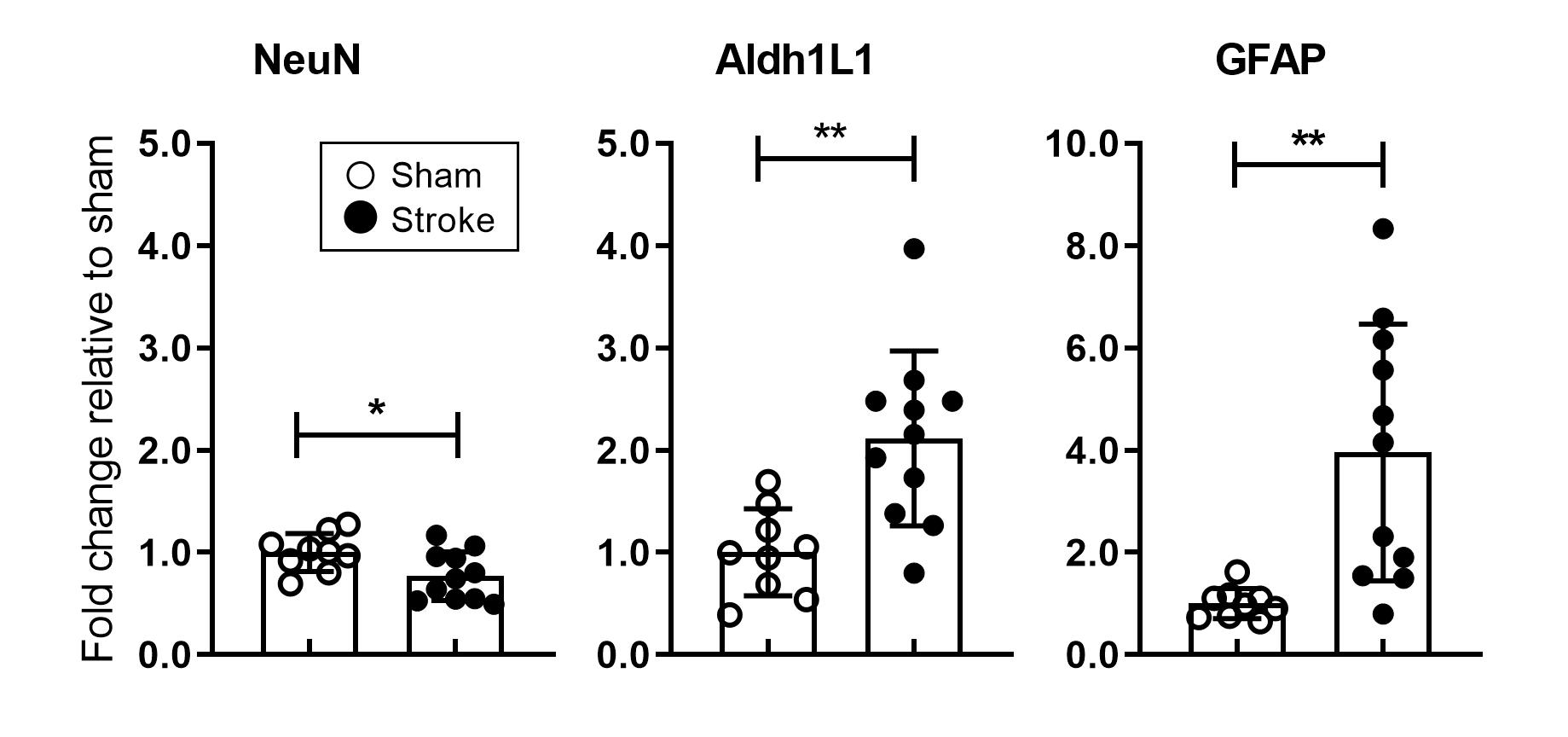


**Supplementary Fig. S2.** **Cortical stroke leads to persistent neuronal loss and astrogliosis within the peri-infarct region.** Western blotting quantification of NeuN, Aldh1L1 and GFAP levels. The protein markers were normalised to β-actin (as loading control), and were expressed as a fold change of mean ± SD relative to the mean of the sham group (sham=9, stroke=11). For full immunoblots, see Supplementary Fig. S4 and S5. p<0.05 (*) and p<0.01 (**)


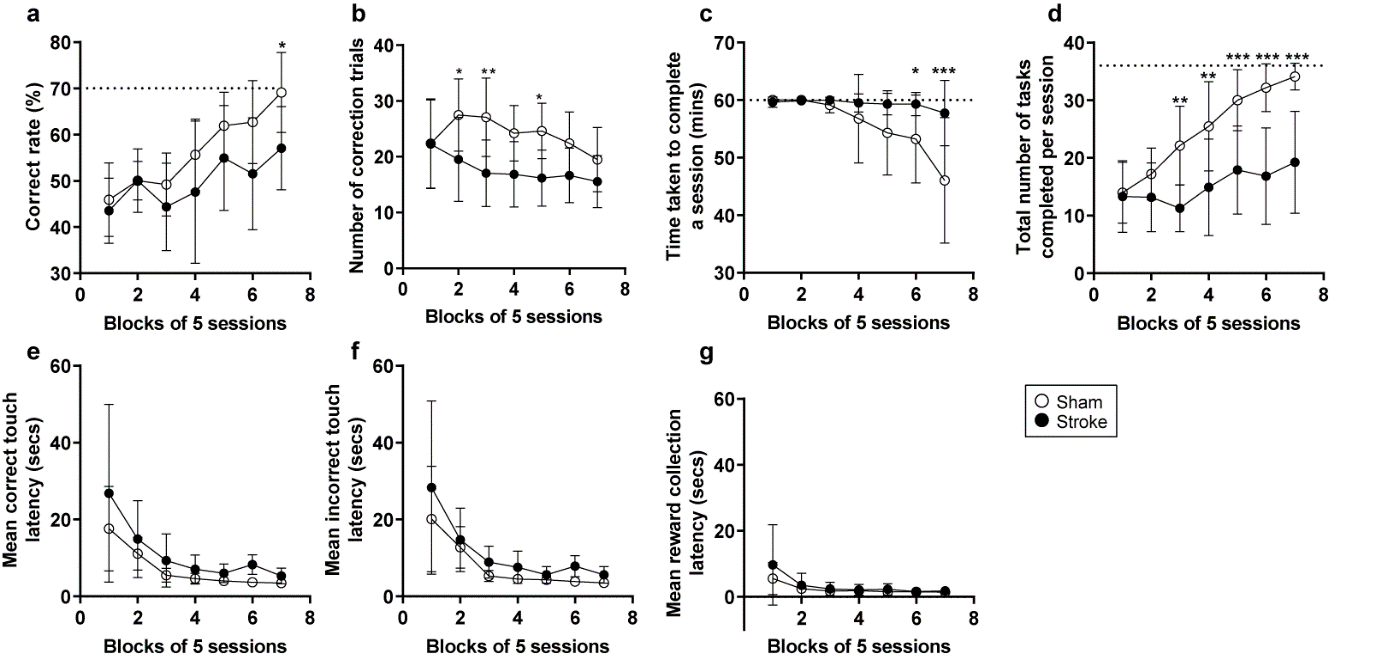


**Supplementary Fig. S3.** **Cognitive assessment in a mouse photothrombotic stroke model using the rodent touchscreen object-location paired-associates learning (PAL) task. a**. Correct rate (%). Minimum correct rate (70 %) as a criterion for acquiring the task was indicated in dotted lines. **b.** Total number of correction trials. **c.** Time taken to complete a session (mins). The maximum time per session (60 mins) was indicated in dotted lines. **d.** Total number of tasks completed per session. The maximum number of tasks which can be completed per session (36 tasks) was indicated in dotted lines. **e**. Mean correct touch latency. **f**. Mean incorrect touch latency. **g**. Mean reward collection latency. Data were presented in mean ± SD (secs) representing an average of five consecutive sessions per block (sham=9, stroke=11). p<.05 (*), p<.01 (**) and p<.001 (***) values were indicated where applicable.


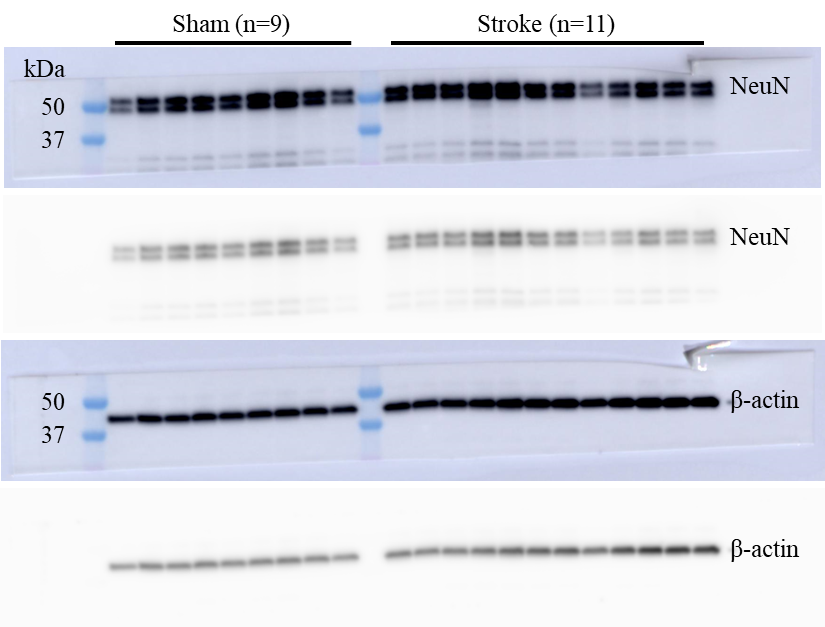


**Supplementary Fig. S4. Immunoblot probed with NeuN, and then stripped and re-probed with β-actin.** Lane 1 and 11 were loaded with molecular weight markers, lane 2 to 10 were loaded with sham samples, and lane 12 to 23 with stroke samples. Top panels were images taken by Amersham Imager 600 for bright field and chemiluminescent overlay, which would provide information of the molecular weight/size of the bands. Bottom panels were chemiluminescent signals for NeuN (~50 kDa) and β-actin (42 kDa). The signals of the bands from the original, unprocessed immunoblots were measured using Amersham Imager 600 Analysis Software, and NeuN was normalised with β-actin. Data was presented in Supplementary Fig. S2.


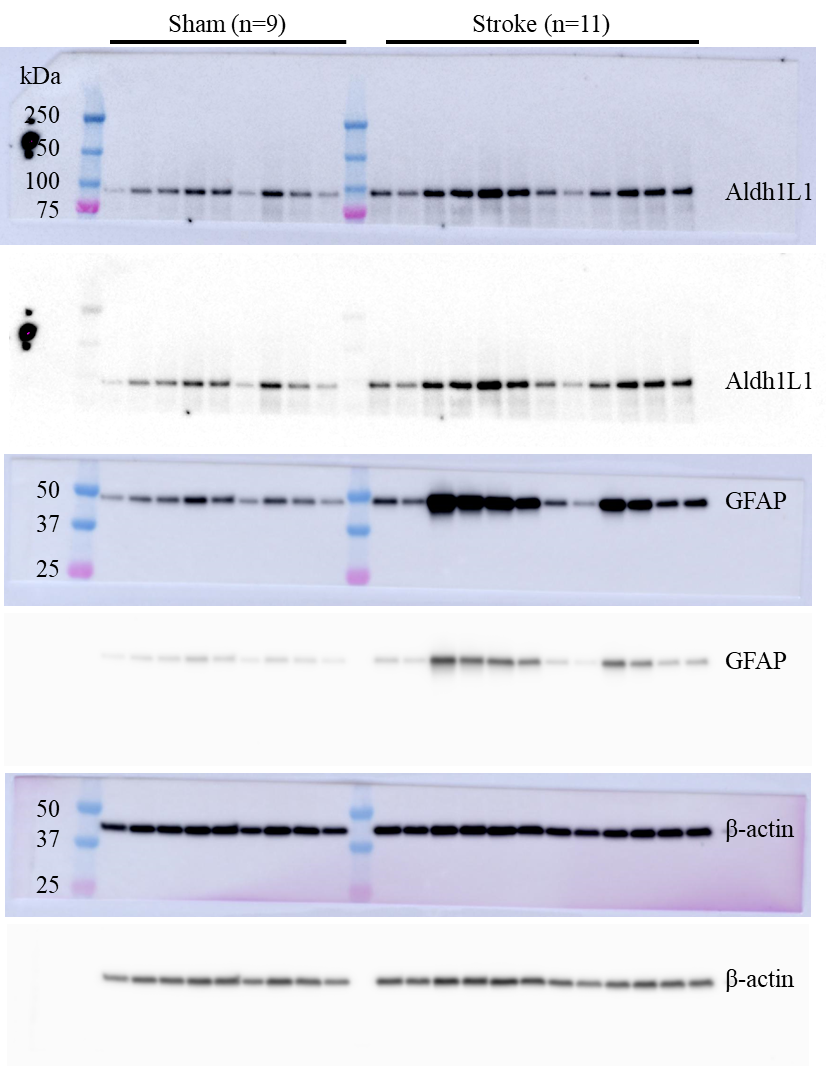


**Supplementary Fig. S5. Immunoblots probed with Aldh1L1 or GFAP. Bottom blot (GFAP) was stripped and re-probed with β-actin.** Lane 1 and 11 were loaded with molecular weight markers, lane 2 to 10 were loaded with sham samples, and lane 12 to 23 with stroke samples. Top panels were images taken by Amersham Imager 600 for bright field and chemiluminescent overlay, which would provide information of the molecular weight/size of the bands. Bottom panels were chemiluminescent signals for Aldh1L1 (100kDa), GFAP (50 kDa), and β-actin (42 kDa). The signals of the bands from the original, unprocessed immunoblots were measured using Amersham Imager 600 Analysis Software, and Aldh1L1 or GFAP was normalised with β-actin. Data was presented in Supplementary Fig. S2.

**References:**

1 Ong, L. K. *et al.* Growth Hormone Improves Cognitive Function After Experimental Stroke. *Stroke* **49**, 1257-1266, doi:10.1161/STROKEAHA.117.020557 (2018).

2 Sanchez-Bezanilla, S., TeBay, C., Nilsson, M., Walker, F. R. & Ong, L. K. Visual discrimination impairment after experimental stroke is associated with disturbances in the polarization of the astrocytic aquaporin-4 and increased accumulation of neurotoxic proteins. *Exp Neurol* **318**, 232-243, doi:10.1016/j.expneurol.2019.05.001 (2019).
